# Evaluation of Antibiotic Use in Patients Admitted to a Hungarian Intensive Care Unit with Pneumonia and Sepsis: Retrospective Observational Before–After Study

**DOI:** 10.3390/antibiotics15030252

**Published:** 2026-02-28

**Authors:** Adina Fésüs, Zsanett Szilágyi, Zsuzsa Beniczky, Eszter Varga, Mária Matuz, Krisztina Gaál, Sándor Somodi, Ildikó Bácskay, István Lekli, Attila Vaskó

**Affiliations:** 1 Department of Pharmacodynamics, Faculty of Pharmacy, University of Debrecen, H-4032 Debrecen, Hungary; 2 Doctoral School of Medical Sciences, University of Debrecen, H-4032 Debrecen, Hungary; 3 Doctoral School of Pharmaceutical Sciences, University of Debrecen, H-4032 Debrecen, Hungary; 4 Institute of Clinical Pharmacy, Faculty of Pharmacy, University of Szeged, H-6725 Szeged, Hungary; 5 Institute of Clinical Pharmacy, Albert Szent-Györgyi Health Center, University of Szeged, H-6725 Szeged, Hungary; 6 Department of Internal Medicine, Faculty of Medicine, University of Debrecen, H-4032 Debrecen, Hungary; 7 Department of Emergency Medicine, Faculty of Medicine, University of Debrecen, H-4032 Debrecen, Hungary; 8 Department of Pharmaceutical Technology, Faculty of Pharmacy, University of Debrecen, H-4032 Debrecen, Hungary; bacskay.ildiko@pharm.unideb.hu; 9 Department of Pulmonology, Faculty of Medicine, University of Debrecen, H-4032 Debrecen, Hungary; vasko.attila@med.unideb.hu

**Keywords:** antibiotic, guideline-adherent, length of stay, MDR pathogen, pneumonia, sepsis, 30-day mortality

## Abstract

**Background**: Early and adequate empiric antibiotic therapy is essential in the treatment of pneumonia and sepsis and may influence the clinical outcome. **Aims and Objectives**: This retrospective before–after study aimed to appraise the impact of a local Antibiotic Stewardship Program (ASP—written guidelines and antibiotic restriction) on antibiotic (AB) use and clinical outcomes in patients requiring intensive care due to pneumonia and sepsis. **Methods**: This study was conducted as a single-center, retrospective observational study in the intensive care unit (ICU) of a pulmonology department of a tertiary care center. Data were collected for the pre-intervention period between January 2018 and May 2022 and for the ASP period between June 2022 and March 2024. In addition to descriptive statistics and univariable methods, interrupted time series (ITS) analysis was used to assess AB use and length of stay in the ICU before and after ASP implementation, using a segmented linear regression with a fixed breakpoint and continuous (hinge) specification. **Results**: The patients admitted to the ICU with pneumonia and sepsis were mainly men (58/101, 57.4% and 84/128, 65.6%), the need for intensive care increased with age, and most of the patients belonged to 65+ age group in both study phases (69/101, 68.3% and 75/128, 58.6%). The majority of the patients had four or more comorbidities (58/101, 57.4% and 52/128, 40.6%). In-hospital mortality was relatively high (42.6% and 41.4%), with most of the patients losing their lives in the ICU (33/43, 76.7% and 37/53, 69.8%). Significant increase in guideline-adherent agent selection (34.5%) and use of combination therapy (35.0%) was observed, while the use of fluoroquinolones decreased significantly (−31.1%). In the after period, a significant decrease in the number of patients using restricted ABs (−53.3%) was observed. In one-third of these cases (10/34, 29.4% and 16/40, 40%), two to four multidrug-resistant pathogens (MDRs) were detected simultaneously, resulting in a significant increase in direct costs (10.5%) in the ICU. The inappropriate use of AB therapy was relatively low in the presence of MDRs in both phases (2/34, 5.9% and 6/40, 15%). In the ASP period, guideline adherence was associated with slightly better clinical outcomes (30-day mortality: −0.8%; length of stay: −22.6%) in pneumonia and sepsis. The ITS analyses after the ASP implementation showed a weak downward trend and before it a slight increasing trend. **Conclusions**: ASP implementation in the ICU resulted in a significant improvement in the appropriate use of ABs, and guideline adherence led to slightly better clinical outcomes. Our results suggest that ASP may offer improved antimicrobial resistance with a sustained long-term effect.

## 1. Introduction

Pneumonia is one of the most common sources of sepsis and septic shock. This life-threatening infection requires intensive care therapy and is associated with early death and a high mortality rate [1]. Consequently, antibiotic treatment is often initiated empirically. Besides the severity of illness and virulence of causative pathogens, inappropriate initial antibiotic therapy may alter the clinical outcome of sepsis caused by pneumonia. Although the use of antibiotics has significantly reduced morbidity and mortality associated with bacterial infections, it has led to an increase in antimicrobial resistance (AMR). Moreover, the World Health Organization (WHO) estimates that AMR was responsible for 1.27 million deaths globally in 2019 [2]. As a result, the treatment of pneumonia and sepsis has become more difficult, especially when the causative pathogens are multidrug-resistant pathogens (MDRs). Those affected are mainly treated in intensive care units (ICUs), accounting for 5.1% of total hospitalized patients, out of which 20.5% have hospital-associated infections (HAIs) like pneumonia and sepsis. In Hungary, HAIs accounted for approximately 3.8% (*N* = 59,654/year), out of which 13.1% (*N* = 7812) were pneumonia and 4.9% (*N* = 2923) sepsis [3].

Intensive care units (ICUs) provide an ideal environment for the emergence and spread of resistant pathogens. AMR in ICUs is a major threat to patient safety and the efficiency of healthcare systems worldwide. It poses a significant challenge to healthcare professionals working in ICUs, where timely and effective antibiotic treatment is crucial, directly impacting clinical outcomes, mortality rates, and increased healthcare costs [4].

A further challenge is that since 2017, merely 12 ABs agents have received approval, and 10 of these fall under established classes that have a known mechanism of AMR [5]. The most commonly used antimicrobial agent (ceftriaxone) accounts for 10.4% of AMR worldwide [3]. In Hungary, while the sensitivity of *Streptococcus pneumoniae* to ceftriaxone is 98.3% in bloodstream infections, the sensitivity of MDR pathogens typical for the ICU has already declined (e.g., 57.5% for *Klebsiella pneumonia*) [6].

International health organizations agree on the importance of an Antibiotic Stewardship Program (ASP); however, according to a survey conducted by the WHO in 2021, only 33% of 163 countries apply the program’s recommendations [7]. The European Centre for Disease Prevention and Control (ECDC) surveillance report indicates a widespread misuse of antibiotics in Hungary. Even though Hungary is among the lower antibiotic users in the European Union in terms of quantity (with 20.8% of inpatients receiving at least one antimicrobial agent), there was no significant decrease (−0.5%) of antibiotic (AB) consumption in the Hungarian hospital sector in the past 10 years [8,9]. In fact, a marked tendency of increased consumption (0.6%) can be observed in the proportion of ABs reserved for treatment of confirmed or suspected infections due to MDRs. The rate of broad-spectrum antibiotics (particularly quinolones) compared to narrow-spectrum antibiotics is 77.3%, and the seasonal fluctuation is 46.5% (significantly high). This is probably due to the high resistance (e.g., in 2023, *Pseudomonas aeruginosa*: 19.9% for piperacillin/tazobactam and 42.2% for imipenem; *Acinetobacter baumannii:* 80.5% for imipenem, 82.8% for ciprofloxacin, 85.5% for levofloxacin, 74.4% for amikacin, and 1.8% for colistin; *Staphylococcus epidermidis*: 99.3% for penicillin, 63.9% for oxacillin, 52.4% for ciprofloxacin, 52.6% for moxifloxacin, and 45.8% for amikacin; *Enterococcus faecium*: 99.2% for ampicillin, 62.2% for gentamicin, 48.2% for vancomycin, and 47.0% for teicoplanin) reported against MDR pathogens, especially from ICU isolates, which entails the use of reserve antibiotics even in combination [9,10]. This may be explained by the fact that currently, there is no official national ASP strategy in Hungary, resulting in limited data on the clinical outcomes of an ASP in the ICU. However, in June 2022, an Antibiotic Stewardship Program (ASP) was initiated in all inpatient care units of the Clinical Centre, University of Debrecen. The objective of the program was to analyze and control the reasonable and cost-effective use of antibiotics. The ASP comprised written and published antibiotic protocols available for all professionals, restricted antibiotic agents, individual regulation of antibiotic use by infectious disease specialists, elements of infection control, and analysis of antibiotic use. The local ASP guidelines for empirical antibiotic therapies were developed based on the local microbiological resistance map and evidence-based antibiotic use [11].

Previous studies have shown that inappropriate AB treatment in community-acquired pneumonia (CAP) led to longer length of stay (LOS) and higher 30-day mortality rate, placing a heavy burden on hospital costs. At the same time, pneumonia-associated sepsis significantly increases the need for intensive care and the risk of mortality [12,13]. However, the implementation of such ASP resulted in significant improvements in CAP therapy in terms of adherence to guidelines, appropriate antibiotic use, and sequential therapy, and it significantly reduced the total duration of empirical antibiotic therapy and the length of hospital stay [11] and limited the development of unintended consequences such as AMR [14].

To our knowledge, despite the importance and incidence of pneumonia and sepsis, no field studies have been performed in Hungarian ICUs to assess the initiated AB therapies. This retrospective before-and-after study aimed to appraise the impact of local ASP on AB use and clinical outcomes in patients requiring intensive care due to pneumonia-associated sepsis in a Hungarian ICU.

## 2. Results

**Figure 1:**
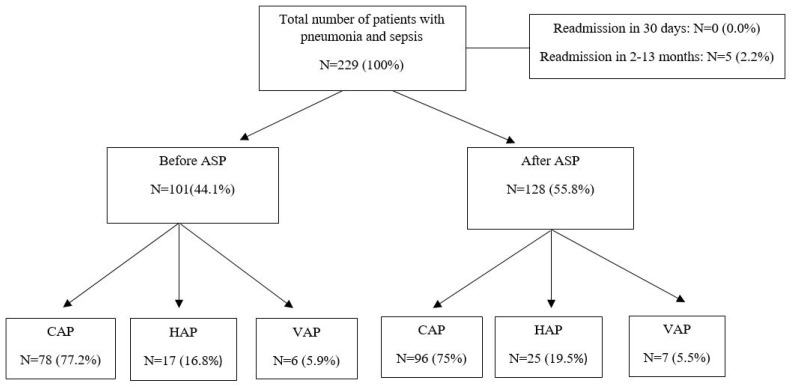
Flowchart for inclusion criteria. ASP: Antibiotic Stewardship Program; CAP: community-acquired pneumonia; HAP: hospital-acquired pneumonia; VAP: ventilator-associated pneumonia.

Data from *N* = 101 and *N* = 128 patients were collected in the before and after phases, respectively (Figure 1). Of them, no patients were excluded since, according to the inclusion criteria, no patients were readmitted within 30 days. Readmission occurred in five cases, but the rehospitalization period was between 2 and 13 months.

### 2.1. Demographic and Clinical Characteristics

The characteristics of patients hospitalized with pneumonia and sepsis are summarized in Table 1. Overall, no significant differences were found regarding patients’ gender and age in the two phases. In both phases, male gender (58/101, 57.4% and 84/128, 65.6%) and patients aged between 65 and 84 years (60/101, 59.4% and 73/128, 57.0%) were predominant. However, age ≥85 years was more frequent in the phase before (9/101, 8.9% vs. 2/128, 1.6%; *p* ˂ 0.05).

Regarding comorbidities, as the CCI increased, the need for intensive care increased proportionally. In both phases, more than half of the patients had a CCI score of 4 or >4 (76/101, 75.2%, and 72/128, 56.2%). Chronic obstructive pulmonary disease and cardiovascular diseases (43/101, 42.6% and 72/128, 56.2%; 62/101, 61.4% and 63/128, 49.2%, respectively) were the most common comorbidities. Moreover, in the phase before chronic kidney/liver diseases occurred more often in the phase before (30/101, 29.7%) than in the phase after (20/128, 15.6%).

No significant differences were found in PSI and CURB-65 scores between the two phases. Nevertheless, in both phases, some patients with lower PSI (˂IV–V) and CURB-65 (˂3–5) scores required intensive care therapy.

### 2.2. Clinical Outcomes

Although the length of stay in the ICU (ICU-LOS) was comparable in both phases (7 and 6.5 days, respectively), the total duration of hospitalization (LOS) was two days shorter in the after phase (14 vs. 12 days).

At the same time, in both phases, one-third of the patients (33/101, 32.7% and 37/128, 29.0%) lost their lives in the ICU, and two-thirds (64/101, 63.4% and 84/128, 65.6%) were discharged from ICU to the pulmonology ward. Furthermore, the in-hospital death rate (43/101, 42.6% and 53/128, 41.4%) was similar to the 30-day mortality rate (43/101, 42.6% and 54/128 42.2%).

### 2.3. Empirical AB Therapy for Pneumonia and Sepsis in the ICU

The characteristics of the first AB therapies in pneumonia and sepsis at admission to the ICU are described in Table 2.

In both phases, combination therapy was the predominant type (59/101, 58.4% and 105/128, 82.0%), while monotherapy decreased by 25.2% in the after phase (from 42/101, 41.6% to 21/128, 16.4%; *p* ˂ 0.05). However, in the first phase, almost half (49.5%) of the combination therapies were intravenous and oral combinations, while in the second phase, their ratio was more than two-thirds (72.7%), and the exclusively intravenously administered AB therapy decreased significantly (by 25.5%, *p* ˂ 0.05).

Regarding guideline adherence, due to the ASP, the guideline-adherent agent selection increased significantly (by 34.5%, from 26/101, 25.7% to 77/128, 60.2%; *p* ˂ 0.05) in the after phase. However, the overall (agent selection, dosage, and duration) guideline adherence similarly occurred in a negligible rate in both periods (1.0% vs. 1.6%).

The appropriate monotherapies were similar in both periods (7/101, 6.9% vs. 8/128, 6.3%), while the guideline-adherent combination therapies increased significantly by 35.0% (from 16/101, 15.8% to 65/128, 50.8%; *p* ˂ 0.001) in the after phase. Furthermore, as a result of the AB restrictions, we found an expressive decrease in the use of fluoroquinolones (by 31.1%, from 33/101, 32.7% to 2/128, 1.6%; *p* ˂ 0.001). Although the unnecessary use of metronidazole occurred at a lower rate in the phase before, it showed a further decrease in the after phase (by 4.3%, from 6/101, 5.9% to 2/128, 1.6%; *p* > 0.05). Other guideline-non-adherent therapies also decreased significantly (by 9.1%, from 21/101, 20.8% to 15/128, 11.7%; *p* ˂ 0.05) except for amoxicillin-clavulanic acid used for CAP therapy, which showed an increase of 4.6% (from 19/101, 18.8% to 30/128, 23.4%; *p* > 0.05).

After the implementation of ASP, the AB exposure showed a slight increase of 10.7% in the ICU (from 15 to 18 DDD/patient) and 8.8% (28 and 27 DDD/patient) during the entire hospitalization. However, the average duration of AB therapies in the ICU decreased by 12.2% and 13.4% during LOS (from a median of 10 to 9 days) in the after phase.

Our further investigations showed a significant decrease in the number of restricted AB users in the after phase (by 53.3% from 76/101, 75.2% to 28/128, 21.9%; *p* < 0.001). As expected, although the use of restricted ABs decreased in the after period (ICU: from median 6.5 to 5 DDD/patient; LOS: from median 10 to 5.5 DDD/patient), the large increase (of 35%) in the use of guideline-adherent AB combinations (Table A1) increased the average costs. The direct AB costs increased significantly both in the ICU and during the entire hospitalization (by 10.5% and 21.4%, respectively; *p* < 0.001).

**Figure 2:**
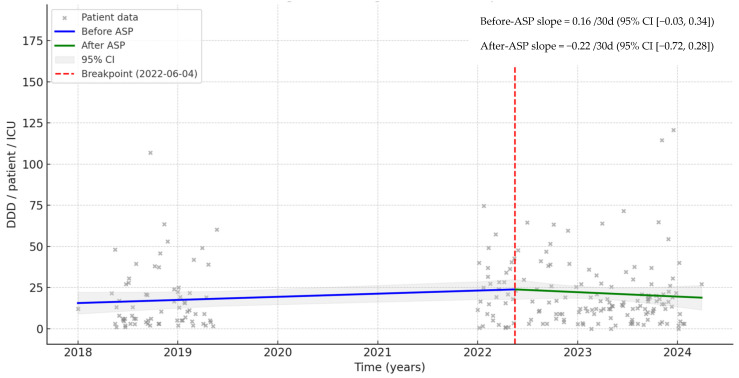
Interrupted time series analysis in the intensive care unit (ICU) before and after implementation of ASP.

**Figure 3:**
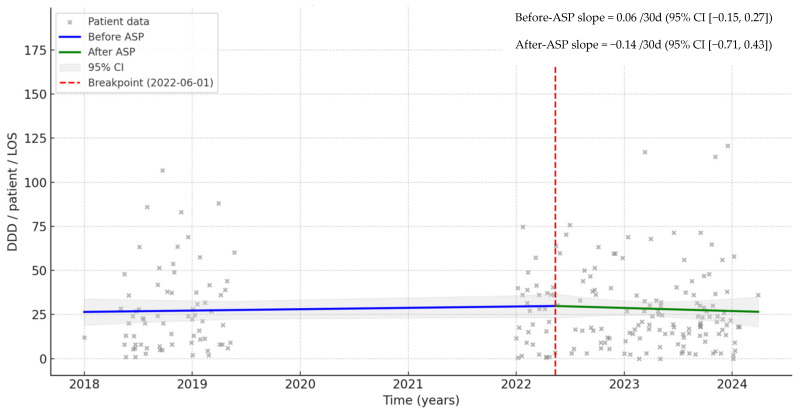
Interrupted time series (ITS) analysis during length of stay (LOS) before and after implementation of ASP.

Despite the pathogen-specific combinations used, the interrupted time series analysis (ITS) indicates a change in the trend of AB use. In the before-ASP phase, the trend increased at approximately 0.16/30 day (95% CI −0.03 to 0.34), while in the after-ASP phase, the trend decreased −0.22/30 day (95% CI −0.72 to 0.28) in the ICU (Figure 2). ITS showed similar results for LOS (Figure 3).

### 2.4. Guideline Adherence and Associated Clinical Outcomes

The comparison of the two phases revealed that guideline adherence resulted in slightly better clinical outcomes in the ICU in pneumonia and sepsis (Table 3). Guideline adherence resulted in a decreased mortality rate (−6.0% vs. −5.5% for in-ICU mortality, −7.2% vs. 6.3% for in-hospital mortality, and −0.8% vs. 2.9% for 30-day mortality) and LOS (−17.6% vs. 0% for ICU-LOS and −22.6% vs. −16.8% for LOS) in the after-ASP period. At the same time, over- or underdosing occurred in both periods. Nevertheless, the frequency remained under 5% for both guideline-adherent and non-adherent agent selection (Table A2).

### 2.5. Multidrug-Resistant Pathogens (MDR) in the ICU

In both phases studied, at least one MDR pathogen was detected in more than one-third of the cases (34/101, 33.6% and 40/128, 31.3%, respectively). Moreover, in one-third of these cases (10/34, 29.4% and 16/40, 40%, respectively) two to four MDR pathogens were detected simultaneously. The most frequently occurring MDRs and their incidence rates during the study are presented in Table 4.

Although there was no significant difference in the frequency of occurrence of MDRs between the two phases, *Pseudomonas aeruginosa*, *Acinetobacter baumannii*, *Staphylococcus* spp., and *Enterococcus faecium* occurred with a higher frequency in the after phase.

Additionally, we found that in both phases, inappropriate AB therapies occurred less frequently in the presence of MDRs (2/34, 5.9% and 6/40, 15%, respectively) than in patients in whom such pathogens were not detected (54/67, 80.6% and 30/88, 34.1%, respectively; *p* < 0.05); nevertheless, in the latter group, the effect of ASP was significant in decreasing inappropriate and restricted AB therapies (Table 5). Similarly, patients with MDRs were exposed to more than twice as many ABs (36.5 and 38 DDD/patient/LOS, respectively) as those without MDRs (14 vs. 18 DDD/patient/LOS, respectively). Although the number of patients with restricted AB treatments decreased significantly in both groups (by 43.9%, from 34/67, 50.7% to 6/88, 6.8%; by 28.2%, from 13/34, 38.2% to 4/40, 10%; *p* < 0.001), the AB exposure increased with the introduction of ASP, regardless of the presence of MDR pathogens, by an average of 4 DDD/patient in the absence of MDR pathogens and by an average of 1.5 DDD/patient in the presence of MDR pathogens during the entire hospitalization. Regarding clinical outcomes, in the presence of MDR pathogens, ICU-LOS was three times longer (median 15 and 13 vs. 4 and 5 days, respectively), while LOS was two times longer (median 20.5 and 17 vs. 11 and 9.5 days, respectively) than in patients without MDR pathogens. Finally, both in-hospital and 30-day mortality rates were higher in the presence of MDRs (one-third and two-thirds of the patients, respectively), but there was no difference between the two phases (Table 5).

## 3. Discussion

Our research aimed to address the following key questions: Can ASP optimize antibiotic use in the ICU without focusing solely on limited antibiotic use and reducing overall antibiotic exposure? How does ASP affect clinical outcomes in pneumonia and sepsis?

In the present study, ASP implementation in the ICU resulted in a significant improvement in the appropriate use of ABs and a significant decrease in the use of restricted ABs, while guideline adherence was accompanied by slightly better clinical outcomes (30-day mortality and LOS).

Antimicrobial resistance continues to represent a critical global health challenge [15]. Administration of prompt and appropriate ABs is an essential element of severe pneumonia and sepsis treatment. ASP implementation in ICUs reported high clinician compliance and significant improvement in AB appropriateness, reducing consumption without compromising patient safety [16,17].

Our results showed that the patients admitted to the ICU with pneumonia and sepsis were mainly men (58/101, 57.4% and 84/128, 65.6%, respectively), the need for intensive care increased with age, and most of the patients belonged to the 65+ age group in both study phases (69/101, 68.3% and 75/128, 58.6%, respectively), strengthening data from the literature [18,19]. The majority of the patients had four or more comorbid conditions (58/101, 57.4% and 52/128, 40.6%, respectively), confirming the impact of chronic diseases as a risk factor for pneumonia and sepsis [1,19]. In terms of clinical outcomes, in-hospital mortality was relatively high (43/101, 42.6% and 53/128, 41.4%), with most of the patients losing their lives in the ICU (33/43, 76.7% and 37/53, 69.8% respectively).

According to a retrospective observational study from 16 general ICUs in the UK, comorbidities affect two-fifths of ICU admissions and can significantly alter disease outcome [20]. Moreover, the presence of pneumonia in patients with severe COPD and cardiac comorbidities was strongly correlated with increased risk for intensive care and high risk of ICU mortality, in accordance with our findings [21]. At the same time, a higher number or severity of existing conditions elevates overall risk, frailty, and the chance of complications, even when considering the acute illness itself. Our results are also supported by a retrospective cohort study of patients admitted with sepsis (61% respiratory site of infection) to 261 ICUs in the UK, which found that in-hospital mortality has decreased in the last 30 years, and it still ranges between 30 and 40% [22].

In our study, ASP implementation with available local protocols for pneumonia and sepsis in the ICU resulted in a significant increase in guideline-adherent agent selection (34.5%) and use of combination therapy (35.0%). Furthermore, restriction in the use of ABs led to a significant decrease in the use of fluoroquinolones (−31.1%) and other (e.g., restricted ABs and their guideline-non-adherent combinations) guideline-non-adherent mono-/combination therapies. In both phases, the high rate (19/101, 18.8% and 30/1288, 23.4%, respectively) of guideline-non-adherent combinations of amoxicillin/clavulanic acid were typically used in patients with low PSI and CURB-65 scores. Although the use of amoxicillin/clavulanic acid has not been recommended in the ICU, this may have occurred since the guideline does not state the therapy for CAP and sepsis. Despite the significant decrease in the number of patients using restricted ABs (−53.3%) and the decreased duration of therapy (by 12.2% in the ICU and by 13.4% during LOS), the average AB exposure increased (by 10.7% in the ICU and by 8.8% during LOS), which resulted in a significant increase in the direct AB costs (by 10.5% in the ICU and by 21.4% during LOS). This can be explained by the use of combinations indicated by local guidelines or by the infectious diseases specialist for pneumonia and sepsis, most often based on the presence of multiple suspected or confirmed MDR pathogens. Although these led to slightly better clinical outcomes, there was no significant difference in LOS or 30-day mortality between the two phases. At the same time, the ITS analysis indicates a decrease in the trend of AB use both for ICU (from 0.16/30 days to −0.22/30 days) and LOS (from 0.06/30 days to −0.14/30 days), suggesting that the ASP may have a sustained long-term effect.

A Canadian retrospective study results show that after implementation of ASP, only 48% of all pneumonia cases in the ICU received guideline-adherent therapy [23]. A systematic review and meta-analysis of 52 studies (1.7 million patients) found that ASP was associated with a 28% decrease in overall AB use (e.g., penicillin and β-lactamase inhibitor combinations by 39%, macrolides by 26%, and cephalosporins by 15%) and a 28% reduction in restricted AB use (e.g., fluoroquinolone by 42% and carbapenems by 31%) [24]. A retrospective Spanish study found that ASP was widely accepted in the ICU and significantly reduced the duration of AB therapy without compromising patient safety [25]. According to a cohort study in Germany, the ASP reduced the duration of AB therapies by 28% and the use of broad-spectrum antimicrobials (e.g., meropenem) in critically ill patients with pneumonia without a negative impact on ICU mortality and ICU-LOS [26]. A retrospective observational study conducted in an ICU setting in Spain showed that the ASP resulted in a decrease in antimicrobial use without a change in clinical outcomes [25]. Moreover, an official American Thoracic Society Workshop Report discussed the opportunities for ASP in pneumonia and sepsis treated in the ICU [27]. According to a Canadian systematic review and meta-analysis of 11 studies conducted in the ICU setting, ASP was not associated with a change in ICU mortality (relative risk for ICU mortality: 1.03, 95% CI 0.93–1.14) [28]. Furthermore, a review of several studies up to 2015 found no convincing evidence for the main purpose of ASP: improving patient outcomes [29]. Although studies investigating the effect of ASP in the ICU on patients with pneumonia and sepsis were hardly found, the aforementioned reviews in the general ICU field are consistent with our overall findings.

In our study, the incidence rate of MDR *Pseudomonas aeruginosa* and *Acinetobacter baumannii* almost doubled compared to the before phase (7/34, 20.6% vs. 15/40, 37.5% and 6/34, 17.6% vs. 14/40, 35%). At the same time, the inappropriate AB therapy was relatively low in the presence of MDRs in both phases (2/34, 5.9% and 6/40, 15%, respectively). The effect of pathogen-specific guidelines developed based on the type of pneumonia and AB restriction was highly demonstrated in cases without MDRs, where a significant reduction in inappropriate AB use (from 54/67, 80.6% to 30/88, 34.1%, *p* < 0.05) was observed. However, the ASP long-term effect could probably have been enhanced by the involvement of an infectious disease specialist, even with restricted AB use, which significantly decreased in both groups (no MDRs: by 43.9%; present MDRs: by 28.2%, respectively; *p* < 0.001). As expected in the presence of MDRs, in both phases, there was an increase in AB exposure and LOS as well as a relatively high in-hospital mortality (22/34, 64.7% and 23/40, 57.5%), which is not surprising, especially when multiple pathogens are present simultaneously. However, the decrease in the use of restricted ABs in the ICU was not associated with worse clinical outcomes.

Previous studies found that the COVID-19 pandemic has permanently changed healthcare, temporarily increasing the rates of HAIs and MDR pathogens worldwide [30,31]. It is not surprising that the presence of MDR bacteria is a serious problem in ICUs, and it is associated with high mortality rates in pneumonia and sepsis. Several studies have found that the most common MDR pathogens in the ICU are *Acinetobacter baumannii*, *Klebsiella pneumoniae*, and *Pseudomonas aeruginosa*, with a relatively high resistance level (over 85%) for ABs, and they present an independent predictor of death [32,33]. In a retrospective cohort study on a large U.S. database, the prevalence of MDR-*Acinetobacter baumannii* was >80%, increasing the risk of receiving inappropriate empiric AB therapy more than fivefold and leading to doubled hospital mortality [34]. According to Spanish national surveillance in the ICU, even with combination therapy, there was only a 30% chance of having an insufficient empirical treatment for a *Pseudomonas aeruginosa* infection [35]. On the other hand, studies show that optimal ASP should not focus solely on decreasing AB use through related costs and increasing guideline adherence but rather on improving individualized AB therapy that may enhance clinical outcomes as well [27,36].

## 4. Strengths and Limitations

Manual data collection allowed for first-hand observation of antibiotic use in the ICU. However, retrospective data collection from medical records may contain inaccurate information or biases resulting from inadequate coding in electronic medical records. A major limitation of the current study was that it was a single-center study with a limited number of patients. A second limitation was that in the before-ASP phase, ICU routine was disrupted by the COVID-19 pandemic, which hampered data collection. Furthermore, the pandemic also acted as a hindering factor, preventing universal adherence to preventive programs and leading to a significant increase in HAIs and the simultaneous spread of MDRs. In light of these, COVID-19 as a confounder could have influenced the comparison between the before-ASP and after-ASP periods.

Third, the DDD is useful for assessing the effectiveness of ASPs; however, there are no DDDs that have been adapted for diseases of specific populations (e.g., pneumonia and sepsis), presenting important methodological limitations in the ICU. Since WHO-defined DDD values are based on average maintenance doses in stable adult populations, DDD-based calculations may overestimate actual antimicrobial exposure and do not reflect patient-level dose adjustments. Therefore, this limitation should be considered when interpreting our findings on antimicrobial consumption. Perhaps when combined with the duration of the AB therapy (DOT) we also use, more representative conclusions can be drawn.

Furthermore, cost inflation could not be accurately tracked, as the study spanned 6.5 years, during which prices may have changed several times.

Finally, the lack of a mortality difference in clinical outcomes may misleadingly suggest the inadequate efficacy of ASP. However, the fundamental goal of ASP is to optimize antimicrobial prescribing practices, including improving guideline adherence, reducing unnecessary antimicrobial exposure, and mitigating antimicrobial resistance. As a result, significant improvements achieved by ASP are more readily observed in prescribing behavior and resistance patterns than in survival. Moreover, mortality in critically ill populations is determined by several factors (e.g., severity of illness, presence of comorbidities and organ failure, etc.) that go far beyond AB treatment. Because these determinants have a significant impact on clinical outcome, ASP interventions represent only a small part of the overall mortality risk profile in the ICU. Accordingly, the absence of a difference in mortality does not reduce the clinical relevance of the ASP; the program’s value is primarily reflected in improved AB use and resistance trends.

## 5. Materials and Methods

### 5.1. Study Design

This study was conducted as a single-center, retrospective observational study in the ICU of a pulmonology department of a tertiary care center in Hungary. In June 2022, the center launched an Antibiotic Stewardship Program (ASP) aimed at regulating AB use.

The latter was conducted by the multidisciplinary antibiotic stewardship team (AST) consisting of pulmonologists, microbiologists, infectious disease specialists, and pharmacists. Antibiotic protocols, restricted (controlled) antibiotic agents, regulation of antibiotic use at the patient’s individual level by infectious disease experts, infection control components, and antibiotic usage analysis were all covered in the ASP guidelines. The local microbiological resistance map and evidence-based antibiotic use served as the basis for developing guidelines for the empirical treatment of sepsis and pneumonia. In addition to the regulation of AB use and infection control measures, adherence to written guidelines available for managing pneumonia and sepsis in the ICU was highly encouraged (Table A1). Furthermore, the guidelines covered in detail the empirical AB therapy of community-acquired pneumonia (CAP), hospital-acquired pneumonia (HAP), and ventilator-associated pneumonia (VAP), with particular attention to AB agents that may be considered in case of suspicion of emerging pathogens (even MDRs), as well as HAP/VAP sepsis but not for sepsis due to CAP. Appropriate dose adapted to the site and type of infection and the optimal therapeutic duration were also included. The preferred mode of administration was intravenous and mainly in combination therapy. Once clinical stability was established, switching from intravenous to oral therapy could be considered [37]. Additionally, a list of restricted ABs (intravenous fosfomycin, ofloxacin, ciprofloxacin, levofloxacin, moxifloxacin, cefiderocol, ceftaroline, ceftazidime/avibactam, ceftolozane/tazobactam, imipenem/cilastatin, meropenem, imipenem/cilastatin/relebactam, meropenem/vaborbactam, linezolid, tedizolid, aztreonam, daptomycin, and colistin) was created to lower the emergence of resistance spreading and guarantee responsible use [38]. The use of these antibiotics was permitted only with the approval of an infectious disease specialist. The pulmonologist completed an electronic approval request form and forwarded it to the infectious disease specialist. Patient information, the required agent (based on pathogen and sensitivity test results or the pulmonologist’s decision if no clinical improvement was found after a microbiological test was available), the first empirical antibiotic therapy, the current clinical outcomes, and the pulmonologist’s contact details were all required. Only the clinical pharmacist, under tight supervision and with the consultant infectologist’s consent, was allowed to dispense restricted antibiotics [11,37,38]. Data collection for the before-ASP phase included patients’ files from 1 January 2018 to 31 May 2022, while the data for the after-ASP phase included the period from 1 June 2022 to 28 March 2024. The study received ethics approval from the Regional Institutional Research Ethics Committee, Clinical Center, University of Debrecen (DE RKEB/IKEB: 6267-2022).

### 5.2. Inclusion and Exclusion Criteria for the Study

All adult patients treated primarily with empirical ABs in the ICU due to pneumonia and sepsis were included in the study. Patients admitted to the ICU of the Department of Pulmonology between 1 January 2018 and 28 March 2024 who were coded with pneumonia and sepsis were identified through electronic records (UD MED System, hospital ward module, Debrecen, Hungary). Patients readmitted for recurrent pneumonia or sepsis within 30 days were excluded from the study.

### 5.3. Data Collection

All patient data and therapy-related information were collected manually from medical records (medication charts, UD-MED Hospital Information System, IT Services Hungary) by pharmacists and pharmacists’ students through predesigned data collection forms. Data collection included demographic and clinical characteristics (gender, age, body weight and height, allergies, comorbidities, admission/discharge type and date, previous hospitalization, and pneumonia/sepsis onset), signs and symptoms (chills/fever, type of cough, absence/presence of sputum, respiratory/heart rate, chest pain, breathlessness, malaise, confusion, blood pressure, dehydration, etc.) at admission in the ICU, chest X-ray results, laboratory test results (arterial pH, blood urea nitrogen, sodium, glucose, hematocrit, partial pressure of oxygen, respiratory rate, white blood cells, C-reactive protein, procalcitonin, interleukin-6, creatinine, estimated glomerular filtration rate, lactase dehydrogenase enzyme, microbiological tests, present pathogens during hospitalization, and antimicrobial susceptibility testing following EUCAST standards), AB therapy during hospitalization (type, agents, mode of administration, dosage, and duration) clinical outcomes (30-day survival and length of stay—LOS). Data on the presence of invasive devices (arterial cannula or bladder catheter) and any oxygen therapy (non-invasive or invasive oxygen supplementation types) were also collected. Finally, all data were recorded in a Microsoft Excel spreadsheet for further analysis. Confidentiality was maintained by anonymization.

### 5.4. Data Analysis and Main Outcome Measures

In this study, we established two phases: “before” and “after” the ASP implementation. Data obtained in both phases were analyzed separately and finally compared with the aim to evaluate the impact of ASP on AB use and clinical outcomes in the ICU.

Charlson Comorbidity Index (CCI) was used to predict 10-year survival in patients with multiple comorbidities [39]. CURB-65 (Confusion–Urea–Respiratory rate–systolic Blood pressure–age; NICE—National Institute for Health and Care Excellence guideline) and PSI (Pneumonia Severity Index) scores were used to estimate the mortality in adult patients with pneumonia and the need for intensive care [40,41]. As stated by the local guideline, patients with a CURB-65 score of 3–5 or PSI score IV–V require ICU admission [11].

The following parameters were compared: demographic and clinical characteristics, comorbidities, CCI, PSI and CURB-65 scores, clinical outcomes (ICU-LOS, LOS, and 30-day mortality), first empirical AB therapy in the ICU (type of therapy: mono- or combination therapy, agent selection, route of administration, dose, duration, or switching from intravenous to oral administration), need for agent change or dose adjustment, AB exposure, guideline adherence, and direct AB costs. In addition, guideline adherence, AB exposure, and clinical outcomes in the presence of MDR pathogens during intensive care were also compared. AB exposure (DDD/patient) was calculated using the ATC/DDD index (version 2024) of the World Health Organization [42], where DDD (defined daily dose) is the estimated average maintenance dose per day for an AB agent used for its main indication in adults. Direct AB costs were calculated based on current purchase prices of the central hospital pharmacy and expressed in HUF/patient.

A pathogen was considered to be a multidrug-resistant (MDR) pathogen when, based on an antibiogram, it was resistant to three or more classes of antimicrobial drugs.

The AB therapy initiated without any microbiological test result but considering suspected pathogens (even MDR) was defined as empirical AB therapy. The first empirical AB therapy in the ICU was considered guideline-adherent only when all selected agents were in concordance with the guideline regarding the type of pneumonia and suspected pathogen (Table A1) [37]. When not all of the antibacterial agents in the combination were adherent, the combined therapy was deemed to be guideline-non-adherent. ICU-LOS and LOS indicated the number of days that the patients spent in the ICU and total hospitalization, respectively.

### 5.5. Statistical Analysis

Fisher’s exact test and two-way ANOVA were applied for comparison of categorical variables, while the Mann–Whitney test was used to compare continuous variables between the two study periods. An interrupted time series (ITS) analysis was used to assess AB use patterns in the ICU before and after ASP implementation. Segmented linear regression (DDD/patient/ICU and DDD/patient/LOS) with a fixed breakpoint (June 2022, ASP implementation) and a continuous (hinge) specification was applied, allowing for a change in slope but not an immediate level change at the intervention date. This approach was chosen because the effect of the ASP was expected to be gradual rather than instantaneous. *p*-values below 0.05 were defined as significant.

## 6. Conclusions

Evaluating AB use before and after the implementation of the ASP helps elucidate the trends of AB use. Early interventions and restrictions may help fight against AB resistance. ASP implementation in the ICU resulted in a significant improvement in the appropriate use of ABs, a significant reduction in the use of restricted ABs (even in the presence of MDR pathogens), and a slight decrease in the AB exposure and duration of AB therapy. ASP had no negative impact on clinical outcomes in pneumonia and sepsis; moreover, it leads to a more coordinated and multidisciplinary effort to improve AMR with a sustained long-term effect, maintaining the efficacy of current antimicrobial agents. Furthermore, our study results encourage the future validation of pneumonia guidelines in the ICU, mainly for CAP and sepsis.

## Figures and Tables

**Table 1:** Demographic and clinical characteristics of the patients in the ICU.

Parameters/Number of Patients	Before ASP *N* = 101	After ASP *N* = 128	*p*-Value
Gender (male)	58 (57.4%)	84 (65.6%)	0.590
Age (years)			
20–64	32 (31.7%)	53 (41.4%)	0.368
65–84	60 (59.4%)	73 (57.0%)	0.913
≥85	9 (8.9%)	2 (1.6%)	0.026 *
CCI score			
0	5 (5.0%)	9 (7.0%)	0.591
1	5 (5.0%)	8 (6.3%)	0.780
2	6 (5.9%)	14 (11.0%)	0.248
3	9 (8.9%)	25 (19.5%)	0.063
4	18 (17.8%)	20 (15.6%)	0.727
>4	58 (57.4%)	52 (40.6%)	0.163
Comorbidities			
Chronic obstructive pulmonary disease	43 (42.6%)	72 (56.2%)	0.248
Cardiovascular disease	62 (61.4%)	63 (49.2%)	0.372
Diabetes mellitus	28 (27.7%)	35 (27.3%)	1.000
Chronic liver/kidney disease (moderate to severe)	30 (29.7%)	20 (15.6%)	0.043 *
Peptic ulcer disease	12 (11.9%)	10 (7.8%)	0.377
Dementia	10 (9.9%)	7 (5.5%)	0.314
Solid tumor			
Localized	10 (9.9%)	9 (7.0%)	0.483
Metastatic	1 (1.0%)	4 (3.1%)	0.390
Cerebrovascular accident or transient ischemic attack	3 (3.0%)	4 (3.1%)	1.000
Peripheral vascular disease	4 (4.0%)	1 (0.8%)	0.176
Hematologic malignancies	1 (1.0%)	3 (2.3%)	0.633
Discharge types			
Moved to the pulmonology ward	64 (63.4%)	84 (65.6%)	0.915
Moved to another hospital ward	3 (3.0%)	3 (2.3%)	1.000
Moved to another ICU	1 (1.0%)	4 (3.1%)	0.390
Clinical outcomes			
In-ICU mortality	33 (32.7%)	37 (29.0%)	0.682
In-hospital mortality	43 (42.6%)	53 (41.4%)	1.000
30-days mortality	43 (42.6%)	54 (42.2%)	1.000
ICU-LOS (means ± SD, median days)	10.20 ± 11.92 (7)	8.88 ± 8.87 (6.5)	0.339
LOS (means ± SD, median days)	17.83 ± 14.93 (14)	15.75 ± 14.95 (12)	0.298
PSI score			
Risk class I (point 0)	-	-	-
Risk class II (points < 70)	5 (5.0%)	12 (9.4%)	0.313
Risk class III (points 71–90)	8 (7.9%)	30 (23.4%)	0.007 *
Risk class IV (points 91–130)	45 (44.6%)	43 (33.6%)	0.314
Risk class V (points > 130)	43 (42.6%)	43 (33.6%)	0.376
CURB-65 score			
Low severity (point 0)	12 (11.9%)	23 (18.0%)	0.359
Moderate severity (points 1–2)	64 (63.4%)	70 (54.7%)	0.514
High severity (points 3–4)	25 (24.8%)	35 (27.3%)	0.771

ICU: intensive care unit; ASP: Antibiotic Stewardship Program; CCI: Charlson Comorbidity Index; LOS: length of stay; SD: standard deviation; PSI: Pneumonia Severity Index; CURB-65: Confusion, Urea, Respiratory rate, and Blood pressure score; * *p* < 0.05.

**Table 2:** Characteristics of empirical antibacterial therapy in the ICU.

Parameters/Number of Patients	Before ASP *N* = 101	After ASP *N* = 128	Increase /Decrease %	*p*-Values
Type of the first AB therapy in the ICU				
Monotherapy	42 (41.6%)	21 (16.4%)	−25.2	0.002 *
Combination therapy	59 (58.4%)	105 (82.0%)	23.6	0.119
No AB therapy #	0 (0.0%)	2 (1.6%)	2.0	0.506
Route of administration				
iv	51 (50.5%)	32 (25.0%)	−25.5	0.007 *
iv + po	50 (49.5%)	93 (72.7%)	23.2	0.084
Guideline-adherent agent(s)	26 (25.7%)	77 (60.2%)	34.5	0.002 *
Guideline-adherent agent(s) and route of administration	10 (9.9%)	16 (12.5%)	2.6	0.839
Guideline-adherent agent(s), route of administration, and dose	6 (5.9%)	13 (10.2%)	4.3	0.342
Guideline-adherent agent(s), route of administration, dose, and duration	1 (1.0%)	2 (1.6%)	0.6	1.000
Guideline-adherent Abs				
Cefepime/ceftazidime/piperacillin-tazobactam/ertapenem/moxifloxacin	7 (6.9%)	8 (6.3%)	−0.6	1.000
Ceftriaxone/cefepime/ceftazidime/piperacillin-tazobactam + clarithromycin/doxycycline/linezolid	16 (15.8%)	65 (50.8%)	35.0	<0.001 **
Other mono/combination therapies	3 (3.0%)	4 (3.1%)	0.1	1.000
Guideline-non-adherent ABs	75 (74.3%)	49 (38.3%)	−36.0	0.008 *
Amoxicillin/clavulanic acid mono/combination therapy	19 (18.8%)	30 (23.4%)	4.6	0.529
Fluoroquinolones mono/combination therapy	33 (32.7%)	2 (1.6%)	−31.1	<0.001 **
Metronidazole mono/combination therapy	6 (5.9%)	2 (1.6%)	−4.3	0.145
Other ^1^ mono/combination therapy	21 (20.8%)	15 (11.7%)	−9.1	0.002 *
Duration of the AB therapy				
ICU (mean ± SD, median days)	8.79 ± 9.37 (6)	7.72 ± 7.12 (6)	−12.2	0.555
LOS (mean ± SD, median days)	12.80 ± 10.77 (10)	11.09 ± 9.99 (9)	−13.4	0.223
AB exposure				
ICU (mean ± SD, median DDD/patient)	21.84 ± 19.95 (15)	24.18 ± 25.41 (18)	10.7	0.494
LOS (mean ± SD, median DDD/patient)	30.44 ± 23.34 (28)	33.11 ± 27.48 (27)	8.8	0.620
Direct AB cost/patient				
ICU (mean ± SD, median HUF)	54,211 ± 64,840 (25,828)	59,905 ± 15,4026 (21,207)	10.5	0.011 *
LOS (mean ± SD, median HUF)	72,126 ± 76,864 (46,458)	87,579 ± 20,6091 (28,658)	21.4	0.020 *
Restrictive AB exposure	*N* = 76 (75.2%)	*N* = 28 (21.9%)	−53.3	<0.001 **
ICU (mean ± SD, median DDD/patient)	9.26 ± 8.44 (6.5)	9.04 ± 10.09 (5)	−2.4	0.419
LOS (mean ± SD, median DDD/patient	13.36 ± 10.93 (10.16)	13.66 ± 17.47 (5.5)	2.2	0.128
Direct restrictive AB cost/patient				
ICU (mean ± SD, median HUF)	44,413 ± 45,040 (28,407)	74,894 ± 170,510 (16,408)	68.6	0.102
LOS (mean ± SD, median HUF)	59,274 ± 57,553 (39,337)	146,270 ± 32,5681 (21,305)	146.8	0.135

AB: antibiotic; ICU: Intensive Care Unit; ASP: Antibiotic Stewardship Program; # due to death at admission in the ICU; iv: intravenous; po: orally; LOS: length of stay; ^1^ other than amoxicillin/clavulanic acid, fluoroquinolones, and metronidazole; SD: standard deviation; DDD: daily defined dose; HUF: Hungarian forint; * *p* < 0.05; ** *p* < 0.001.

**Table 3:** Guideline adherence and associated clinical outcomes in the ICU.

Parameters	Before ASP	After ASP	Increase/ Decrease %	*p*-Values
Guideline-adherent AB agent(s) *	26 (100%)	77 (100%)	-	-
In-ICU mortality	9 (34.6%)	22 (28.6%)	−6.0	0.382
In-hospital mortality	11 (42.3%)	27 (35.1%)	−7.2	0.328
30-day mortality	10 (38.5%)	29 (37.7%)	−0.8	0.324
ICU-LOS (means ± SD, median days)	12.65 ± 18.94 (8.5)	8.70 ± 7.45 (7)	−17.6	0.349
LOS (means ± SD, median days)	21.81 ± 22.56 (15.5)	17.43 ± 16.67 (12)	−22.6	0.247
Underdosing	5/26 (19.2%)	3/77 (4.0%)	−15.2%	0.204
Overdosing ^1^	1/26 (3.8%)	3/77 (4.0%)	0.2%	0.192
Guideline-non-adherent AB agent(s) *	75 (100%)	49 (100%)	-	-
In-ICU mortality	24 (32.0%)	13 (26.5%)	−5.5	0.882
In-hospital mortality	32 (42.7%)	24 (49.0%)	6.3	0.564
30-day mortality	33 (44.0%)	23 (46.9%)	2.9	0.541
ICU-LOS (means ± SD, median days)	9.35 ± 8.35 (6)	9.45 ± 10.92 (6)	0	0.934
LOS (means ± SD, median days)	16.45 ± 11.17 (14)	13.69 ± 11.76 (11)	−16.8	0.105
Underdosing	7/75 (9.3%)	8/49 (16.3%)	7.0%	0.053
Overdosing ^2^	2/75 (2.7%)	2/49 (4.1%)	1.4%	1.000

AB: antibiotic; ASP: Antibiotic Stewardship Program; ICU: Intensive Care Unit; * first empiric AB therapy in the ICU; LOS: length of stay; SD: standard deviation; ^1^ inappropriate dose due to low creatinine clearance; ^2^ inappropriate dose due to extremely low body weight.

**Table 4:** Most common MDRs in pneumonia and sepsis in the ICU.

Parameters/ Number of Patients	Before ASP *N* = 34/101 (100%)	After ASP *N* = 40/128 (100%)	*p*-Values
*Pseudomonas aeruginosa*	7 (20.6%)	15 (37.5%)	0.327
*Acinetobacter baumannii*	6 (17.6%)	14 (35.0%)	0.308
*Staphylococcus epidermidis*	10 (29.4%)	10 (25.0%)	0.804
*Enterococcus faecium*	7 (20.6%)	7 (17.5%)	0.780
*Staphylococcus haemolyticus*	5 (14.7%)	6 (15.0%)	1.000
*Klebsiella pneumoniae*	3 (8.8%)	6 (15.0%)	0.725
*Staphylococcus hominis*	7 (20.6%)	3 (7.5%)	0.190
*Staphylococcus aureus*	5 (14.7%)	0 (0.0%)	0.026 *
*Streptococcus* species	1 (2.9%)	0 (0.0%)	0.467
*Enterobacter cloacae*	1 (2.9%)	0 (0.0%)	0.467
*Escherichia coli*	1 (2.9%)	0 (0.0%)	0.467

ASP: Antibiotic Stewardship Program; * *p* ˂ 0.05.

**Table 5:** Antibiotic therapies and clinical outcomes in patients with or without MDRs in the ICU.

Parameters/ Number of Patients	No MDRs			Presence of MDRs		
	Before ASP *N* = 101 (100%)	After ASP *N* = 128 (100%)	*p*-Value	Before ASP *N* = 101 (100%)	After ASP *N* = 128 (100%)	*p*-Value
Frequency	67 (66.3%)	88 (68.8%)	0.917	34 (33.7%)	40 (31.3%)	0.789
Inappropriate AB therapy ^1^	54/67 (80.6%)	30/88 (34.1)	0.002 *	2/34 (5.9%)	6/40 (15.0%)	0.456
Restricted AB users–LOS	34/67 (50.7%)	6/88 (6.8%)	<0.001 **	13/34 (38.2%)	4/40 (10.0%)	0.031 *
AB exposure–ICU-LOS (mean ± SD, median DDD/patient)	12.06 ± 15.88 (6)	14.42 ± 11.94 (12)	0.012 *	31.85 ± 17.62 (30)	37.70 ± 36.21 (26)	0.895
AB exposure–LOS (mean ± SD, median DDD/patient)	20.60 ± 20.20 (14)	21.57 ± 16.51 (18)	0.313	38.56 ± 21.03 (36.5)	46.13 ± 37.99 (38)	0.736
ICU-LOS (mean ± SD, median days)	6.48 ± 6.45 (4)	6.18 ± 4.75 (5)	0.742	17.53 ± 16.09 (15)	14.8 ± 12.30 (13)	0.418
LOS (mean ± SD, median-days)	15.34 ± 13.81 (11)	11.57 ± 9.25 (9.5)	0.126	22.74 ± 15.81 (20.5)	24.95 ± 20.10 (17)	0.668
In-hospital mortality	21/67 (31.3%)	30/88 (34.1%)	0.871	22/34 (64.7%)	23/40 (57.5%)	0.850
30-day mortality	22/67 (32.8%)	30/88 (34.1%)	1.000	21/34 (61.8%)	24/40 (60.0%)	1.000

MDRs: multidrug-resistant pathogens; ASP: Antibiotic Stewardship Program; AB: antibiotic; ^1^ the pathogen was resistant to the therapy used, or underdosing/overdosing in case of appropriate agent selection; LOS: length of stay; ICU: Intensive Care Unit; SD: standard deviation; DDD: daily defined dose. * *p* < 0.05; ** *p* < 0.001.

## Data Availability

Data are available from the corresponding author upon reasonable request.

## Abbreviations

The following abbreviations are used in this manuscript:

AB	antibiotic
AMR	antimicrobial resistance
ASP	Antibiotic Stewardship Program
CAP	community-acquired pneumonia
CCI	Charlson Comorbidity Index
CURB-65	score Confusion–Urea–Respiratory rate–systolic Blood pressure–age
DDD	defined daily dose
ECDC	European Centre for Disease Prevention and Control
HAIs	hospital-associated infections
HAP	hospital acquired pneumonia
ICU	intensive care unit
LOS	length of stay
MDRs	multidrug-resistant pathogens
NICE	National Institute for Health and Care Excellence
PSI	Pneumonia Severity Index
VAP	ventilator-associated pneumonia
WHO	World Health Organization

## Appendix A

### Appendix A.1

**Table A1:** Local guideline for empirical antibiotic therapy for pneumonia and sepsis in the ICU.

Diseases	Agents	Dosage		
		Dosage Form	Dose	Duration
CAP	cefotaxime/ ceftriaxone/ *ceftaroline* ^1^ + azithromycin */ clarithromycin/ doxycycline **	iv	4–6 × 2 g 1 × 2 g 2 × 600 mg 1 × 500 mg 2 × 500 mg 2 × 100 mg	5 days
	*levofloxacin/* *moxifloxacin*	iv	2 × 500 mg 1 × 400 mg	5 days
	ceftazidime/ cefepime/ piperacillin-tazobactam + azithromycin */ clarithromycin	iv	3 × 2 g 3 × 2 g 4 × 4.5 g 1 × 500 mg 2 × 500 mg	5–7 days
	azithromycin ^2^ + aztreonam ^2^ + *linezolid* ^2^	iv	1 × 500 mg 3 × 2 g 2 × 600 mg	5 days
Aspiration pneumonia	ampicillin-sulbactam/ amoxicillin-clavulanic acid	iv	4 × 3 g 4 × 1.2 g	5 days
	*moxifloxacin*/ clindamycin	iv	1 × 400 mg 3 × 900 mg	5 days
HAP No suspected MDR	ceftazidime/ cefepime/ piperacillin-tazobactam/ ertapenem	iv	3 × 2 g 3 × 2 g 4 × 4.5 g 1 × 1 g	7 days
	*levofloxacin/* *aztreonam*	iv	2 × 500 mg 3 × 2 g	7 days
HAP MRSA	ceftazidime/ cefepime/ piperacillin-tazobactam/ ertapenem + *linezolid*	iv	3 × 2 g 3 × 2 g 4 × 4.5 g 1 × 1 g 2 × 600 mg	7–10 days
	*levofloxacin/* *aztreonam* + *linezolid*	iv	2 × 500 mg 3 × 2 g 2 × 600 mg	7–10 days
HAP *Pseudomonas aeruginosa*	ceftazidime/ cefepime + amikacin/ tobramycin	iv	3 × 2 g 3 × 2 g 1 × 15 mg/kg 1 × 5–7 mg/kg	7–10 days
	*ciprofloxacin/* *aztreonam* + amikacin/ tobramycin	iv	3 × 400 mg 3 × 2 g 1 × 15 mg/kg 1 × 5–7 mg/kg	7–10 days
HAP MDR *Acinetobacter baumannii*	*colistin* + tigecycline/ ampicillin-sulbactam	iv	LD: 9 mIU, MD: 2 × 4.5 mIU LD: 200 mg, MD: 2 × 100 mg 3 × 9 g	7–10 days
	*colistin* + tigecycline	iv	LD: 9 mIU, MD: 2 × 4.5 mIU LD: 200 mg, MD: 2 × 100 mg	7–10 days
HAP CRE	*ceftazidime-avibactam +/−* *aztreonam/* *colistin*	iv	3 × 2.5 g 3 × 2 g LD: 9 mIU, MD: 2 × 4.5 mIU	7 days
	*colistin* + tigecycline	iv	LD: 9 mIU, MD: 2 × 4.5 mIU LD: 200 mg, MD: 2 × 100 mg	7 days
HAP/VAP + sepsis	cefepime/ *meropenem* + *colistin* + sulfamethoxazole-trimethoprim	iv	3 × 2 g 3 × 2 g LD: 9 mIU, MD: 2 × 4.5 mIU 2 × 10 mg/kg	7–10 days
	*aztreonam* + *colistin* + sulfamethoxazole-trimethoprim	iv iv	3 × 2 g LD: 9 mIU, MD: 2 × 4.5 mIU 2 × 10 mg/kg	7–10 days

Green color: first-line antibiotic; blue color: real severe beta-lactam allergy; orange color: second-line antibiotic, in case of failure of first-line antibiotic; no background color: real sever beta-lactam allergy in sepsis; italic: restricted antibiotics; ICU: Intensive Care Unit; CAP: community-acquired pneumonia; HAP: hospital-acquired pneumonia; MDR: multidrug-resistant pathogens; MRSA: methicillin-resistant *Staphylococcus aureus*; CRE: carbapenem-resistant Enterobacteriaceae; VAP: ventilation-associated pneumonia; iv: intravenously; LD: loading dose; MD: maintenance dose; *: 3 days; **: 7 days; ^1^ in winter, epidemic period-in case of *Staphylococcus aureus*; ^2^ very severe beta-lactam allergy.

### Appendix A.2

**Table A2:** Guideline adherence and associated clinical outcomes in the ICU.

Parameters/ Number of Patients	Before ASP N = 101				After ASP N = 128				Before–After ASP
	Guideline-Adherent * N = 26 (100%)	Guideline-Non-Adherent * N = 75 (100%)	Increase/ Decrease %	*p*- Values	Guideline-Adherent * N = 77 (100%)	Guideline-Non-Adherent* N = 49 (100%)	Increase/ Decrease %	*p*- Values	Increase/ Decrease %
In-ICU mortality	9 (34.6%)	24 (32.0%)	−2.6	0.237	22 (28.6%)	13 (26.5%)	−2.1	0.630	0.622
In-hospital mortality	11 (42.3%)	32 (42.7%)	0.4	0.264	27 (35.1%)	24 (49.0%)	13.9	0.635	0.274
30-day mortality	10 (38.5%)	33 (44.0%)	5.5	0.250	29 (37.7%)	23 (46.9%)	9.2	0.597	0.394
ICU-LOS (means ± SD, median days)	12.65 ± 18.94 (8.5)	9.35 ± 8.35 (6)	−29.4	0.341	8.70 ± 7.45 (7)	9.45 ± 10.92 (6)	−14.3	0.894	0.145
LOS (means ± SD, median days)	21.81 ± 22.56 (15.5)	16.45 ± 11.17 (14)	−9.5	0.485	17.43 ± 16.67 (12)	13.69 ± 11.76 (11)	−8.3	0.257	0.642
Underdosing	5 (19.2%)	7 (9.3%)	−9.9	0.548	3 (4.0%)	8 (16.3%)	12.3	0.811	0.899
Overdosing ^1^	1 (3.8%)	2 (2.7%)	1.1	0.583	3 (4.0%)	2 (4.1%)	0.1	0.772	0.553

ICU: Intensive Care Unit; ASP: Antibiotic Stewardship Program; * first empiric antibiotic therapy in the ICU; LOS: length of stay; SD: standard deviation; ^1^ inappropriate dose due to low creatinine clearance or extremely low body weight.
